# The Role of Enoxaparin in Influenza Virus Infections and its Therapeutic Implications

**DOI:** 10.1093/infdis/jiaf470

**Published:** 2025-09-10

**Authors:** Marta Bermejo-Jambrina, Viktoria Zaderer, Julia Eder, Gabriel Diem, Killian E Vlaming, Wilfried Posch, Teunis B H Geijtenbeek, Doris Wilflingseder

**Affiliations:** Institute of Hygiene and Medical Microbiology, Medical University of Innsbruck, Innsbruck, Austria; Department of Experimental Immunology, Amsterdam University Medical Centers, University of Amsterdam, Amsterdam, The Netherlands; Amsterdam Institute for Infection and Immunity, Amsterdam, The Netherlands; Institute of Hygiene and Medical Microbiology, Medical University of Innsbruck, Innsbruck, Austria; Department of Experimental Immunology, Amsterdam University Medical Centers, University of Amsterdam, Amsterdam, The Netherlands; Amsterdam Institute for Infection and Immunity, Amsterdam, The Netherlands; Department of Dermatology, Medical University of Vienna, Vienna, Austria; Research Center for Molecular Medicine, Austrian Academy of Sciences, Vienna, Austria; Institute of Hygiene and Medical Microbiology, Medical University of Innsbruck, Innsbruck, Austria; Department of Experimental Immunology, Amsterdam University Medical Centers, University of Amsterdam, Amsterdam, The Netherlands; Amsterdam Institute for Infection and Immunity, Amsterdam, The Netherlands; Institute of Hygiene and Medical Microbiology, Medical University of Innsbruck, Innsbruck, Austria; Department of Experimental Immunology, Amsterdam University Medical Centers, University of Amsterdam, Amsterdam, The Netherlands; Amsterdam Institute for Infection and Immunity, Amsterdam, The Netherlands; Ignaz Semmelweis Institute, Interuniversity Institute for Infection Research, University of Veterinary Medicine Vienna, Vienna, Austria

**Keywords:** enoxaparin, influenza, mucosal immunology, inflammation, cytokines

## Abstract

Frequent emergence of respiratory viruses with pandemic potential, like severe acute respiratory syndrome coronavirus 2 or influenza, underscores the need for broad-spectrum prophylaxis. Existing vaccines show reduced efficacy against newly emerged variants, and the ongoing risk of new outbreaks highlights the importance of alternative strategies to prevent infection and viral transmission. As respiratory viruses primarily enter through the nose, formulations targeting the nasal epithelium are attractive candidates to neutralize pathogens and thus prevent or minimize infection. Enoxaparin, a low-molecular-weight heparin (LMWH) widely used as an anticoagulant, also exhibits antiviral and anti-inflammatory properties. We found that in highly differentiated human nasal and upper respiratory tract 3D models, enoxaparin inhibited influenza A/H3N2 and B/Victoria infection, reduced release of proinflammatory cytokines and chemokines, and mitigated epithelial damage caused by infection. Our study highlights the LMWH inhibitory effect on respiratory viruses. When applied to mucosal entry sites, LMWH shows promise as prophylactic and valuable alternative to traditional antiviral approaches.

## Teaser

This study demonstrates that enoxaparin blocks influenza infection and associated inflammation in nasal tissue models, supporting its potential as a mucosal prophylactic against influenza and other respiratory viruses, including SARS-CoV-2.

Pandemic respiratory viruses spread rapidly among immunologically naive populations, with no vaccines available against new strains. In such cases, antiviral prophylaxis treatment represents a valuable option.

The respiratory epithelium is a key entry site for respiratory viruses, such as influenza and severe acute respiratory syndrome coronavirus 2 (SARS-CoV-2), transmitted via droplets and aerosols [1, 2]. Primary infection predominantly occurs in the nasal epithelium [3, 4]. It has been proposed that droplets tend to deposit most densely in the nasal cavity where they lead to infection before spreading to the lower airways [2, 5]. The nasal epithelium plays a crucial role in initiating innate immune responses, including interferon production and immune cell recruitment [4, 6, 7], which are critical in limiting viral replication and spread like influenza and SARS-CoV-2, thus preventing severe outcomes.

To mimic the respiratory epithelium, we used 3D primary epithelial models grown at an air-liquid interface (ALI), which replicate essential cell types (ciliated, goblet, basal cells) and their functions, allowing detailed study of virus–host interactions [8, 9]. Comparisons of nasal and bronchial epithelial cells reveal distinct innate immune responses, with nasal cells mounting stronger reactions, underscoring their frontline role [10, 11].

Recent SARS-CoV-2 studies have underscored the importance of nasal epithelial immunity in disease severity and controlling infection at entry sites [12]. SARS-CoV-2 binds to heparan sulfate proteoglycans (HSPGs), facilitating angiotensin-converting enzyme 2 (ACE2)–mediated entry [13–16]. HSPGs are abundant in the respiratory tract [17]. Low-molecular-weight heparins (LMWHs), like enoxaparin, commonly used as anticoagulants [18, 19], competitively block SARS-CoV-2 binding to HSPGs [14–16, 20], preventing infection and serving as potential prophylactics [14, 16]. However, their effectiveness against other respiratory viruses remains unclear.

Here, we investigated LMWH's impact on influenza A/H3N2 and B/Victoria infection using 3D-human airway epithelial tissue models of the nasal and bronchial epithelium. Enoxaparin treatment was applied, resulting in protection of tissues from viral damage, reduced complement C3 activation, and prevention of cytokine secretion. Phenotypic and genotypic analyses revealed tissue-specific differences in cytokine and chemokine induction. Additionally, ALI models were validated for antiviral drug screening through viral protein immunostaining, fluorescence imaging, and RNA quantification. These findings support enoxaparin as a practical prophylactic to block infection, reduce inflammation, and prevent disease progression.

## MATERIALS AND METHODS

### Study Approval

Clinical virus isolates were obtained from leftover/anonymized nasopharyngeal/oropharyngeal swab specimens and ethylenediaminetetraacetic acid blood collected during routine diagnostics, with written informed consent for research use. These specimens’ use was approved by the Medical University of Innsbruck Ethics Committee (ECS1166/2020).

### Cell Culture

Primary human nasal mucosa and normal human bronchial epithelial (NHBE) cells, available in the laboratory, were routinely cultured in ALI as previously described [9, 21, 22].

For viral propagation, Madin-Darby canine kidney (MDCK) cells were used for influenza and VeroE6-TMPRSS2/ACE2 cells for SARS-CoV-2 (wild-type [WT] and variants of concern [VoCs]). These cells were maintained until infection in Dulbecco's modified Eagle's medium (DMEM) supplemented with 10% fetal calf serum, 1% L-glutamine, and 1% penicillin-streptomycin at 37°C, 5% CO_2_. The high-glucose formulation of DMEM was used for VeroE6 cells. All reagents were from Sigma-Aldrich.

### Compounds

*N*-Acetyl-2,3-dehydro-2-deoxyneuraminic acid (DANA; Sigma-Aldrich) was prepared at 10 mM in bi-distilled water and stored at −20°C. Enoxaparin (Sanofi) was stored at room temperature.

### Enoxaparin Treatment and Infection Assays

To evaluate prophylactic effects, enoxaparin spray (∼50 μL; 23.4 IU/mL/3000 IU/mL) was administered apically 30 minutes before infection with influenza A/H3N2, influenza B/Victoria, and SARS-CoV-2 (WT, VoCs), without epithelial surface disruption. We selected enoxaparin (150 mg/mL) based on previous *in vitro* studies demonstrating effective SARS-CoV-2 inhibition [14, 16]. Enoxaparin concentrations were chosen to remain within a clinically relevant and safe range, based on a prior ethics-approved human trial [16]. Two hours prior to infection, Transwell medium was refreshed with ALI medium (0.7 mL in basolateral chamber). Before infection, enoxaparin was applied apically and incubated at 37°C for 30 minutes. Subsequently, cells were infected with the respective virus. Treatment effects were assessed by comparing transepithelial electrical resistance (TEER) values between enoxaparin-treated and phosphate-buffered saline–treated control cells.

### TEER Measurement

TEER values were measured on ALI cultures using an EVOM volt ohmmeter with STX-2 electrodes (World Precision Instruments). For measurements, prewarmed 0.1 mL medium was added to equilibrate the cells and removed postmeasurement to restore ALI conditions. Values were corrected for Transwell filter resistance.

### Viruses

SARS-CoV-2 WT, influenza A/H3N2, and influenza B/Victoria were obtained from BEI Resources, and SARS-CoV-2 VoCs (Delta [B.1.617.2] and Omicron [BA.5]) were isolated from SARS-CoV-2–positive swabs and cultured [23].

### Real-Time Reverse-Transcription Polymerase Chain Reaction for Viral Quantification and Respiratory Tract Marker Expression

Viral RNA was extracted using the FavorPrep Viral RNA Mini Kit (FAVORGEN), while total RNA for host gene expression was extracted with the peqGOLD total RNA kit (VWR Life Science), per manufacturer instructions. Influenza was detected by targeting the matrix gene (Supplementary Table 1) and SARS-CoV-2 via the N1 region of the nucleocapsid gene (https://archive.cdc.gov/www_cdc_gov/coronavirus/2019-ncov/lab/multiplex.html). Primers were designed with Primer Express 2.0 (Applied Biosystems); sequences are listed in Supplementary Table 1. Real-time reverse-transcription polymerase chain reaction (RT-qPCR) was performed using the Luna Universal Probe One-Step RT-qPCR Kit (NEB) with absolute quantification by standard curves from National Institute for Biological Standards and Control RNA controls. Messenger RNA (mRNA) levels were normalized to household gene (GADPH) with the equation N_t_ = C_t_ (GAPDH) − Ct(target). All reactions were run on the Bio-Rad CFX96 system and analyzed with Bio-Rad CFX Maestro 1.1 software.

### Staining and High-Content Screening

Cells were infected with influenza A/H3N2 or B/Victoria and analyzed 72 hours postinfection (hpi) using high-content confocal microscopy (Operetta CLS, PerkinElmer). Cultures were fixed (4% paraformaldehyde), sectioned from Transwells, and stained intracellularly for influenza nucleoprotein (NP) using conjugated antibodies (Supplementary Table 2) [21, 22]. Stained cultures were mounted in Mowiol and analyzed (∼1200 cells/condition) using Harmony 4.9 software. Ciliated and goblet cells were phenotypically characterized (Supplementary Table 2) and ciliary motion was assessed by tracking bead movement (Supplementary Table 2) [9]. Complex 3D models were profiled phenotypically in a high-throughput manner with Operetta CLS system. Live cell imaging in non-confocal mode assessed ciliary motion and mucociliary clearance. Mucociliary clearance was monitored using the Operetta CLS in a fully automated setup within Harmony 4.9 software by tracking fluorescently labeled beads (Supplementary Table 2) applied apically to ALI tissues, with clearance velocity calculated from bead displacement and speed under different conditions.

### Fluorescence Plaque Assay

MDCK cells (96-WP, 150 000 cells/well) were infected with 10-fold serial dilutions of influenza A/H3N2 and influenza B/Victoria for 1 hour. After virus incubation, cells were washed and media replaced. At 16 hpi, cells were fixed, permeabilized, and stained with influenza NP antibodies, followed by goat-antirabbit immunoglobulin G (Supplementary Table 2).

### Lactate Dehydrogenase Quantification

Cytotoxicity was assessed using Roche’s lactate dehydrogenase (LDH) detection kit following the manufacturer’s instructions. Absolute values were calculated using an L-LDH standard curve of (0.05–10 U/mL; Roche Applied Science), showing linear dynamic range (*r* = 0.996).

### Profiling of Cytokines/Chemokines and Anaphylotoxin C3a and C5a

Supernatants collected at 72 hpi were inactivated with 2% Igepal and analyzed by enzyme-linked immunosorbent assay (ELISA) for interleukin (IL) 6, IL-10, CXCL-10, IL-8, C3a, and C5a secretion (eBiosciences, BD Biosciences) according to the manufacturer's instructions. Optical density at 450 nm values were measured using BioTek Synergy HT.

### Statistical Analysis

Data were analyzed by GraphPad Prism 9 software and are shown as mean ± standard error of the mean. Paired samples were compared by 2-tailed Student *t*-test, and nonparametric unpaired samples by Mann–Whitney test. One-way or 2-way analysis of variance with Tukey, Šídák, or Dunnett post hoc test was used for multiple comparisons. Half-maximal inhibitory concentration (IC_50_) values were determined by nonlinear regression. Statistical significance was set at *P* ≤ .05, *P* ≤ .01, *P* ≤ .001, or *P* < .0001. All experiments were independently repeated at least 3 times and ≥1000 cells were analyzed per image.

## RESULTS

### Characterization and Comparison of Nasal and Bronchial Epithelial 3D Models

*In vitro* cultured nasal epithelial cells are commonly used as surrogates for bronchial epithelium [11], but differences in cellular composition may influence infection and disease progression [24, 25]. To investigate this, nasal and bronchial epithelial cells were differentiated in an ALI model [9]. We first assessed characteristic respiratory epithelial markers: FOXJ1 (ciliated cells), MUC5AC (goblet/secretory cells), and KRT5 (basal cells) 35 days post-ALI (Supplementary Figure 1*A*) and ciliated/goblet cells by immunofluorescence (IF) 50 days post-ALI (Supplementary Figure 1*B* and 1*C*). Nasal epithelial cells showed significantly higher MUC5AC mRNA expression compared to bronchial cell cultures (NHBE), while FOXJ1 and KRT5 mRNA levels were similar in both cell types (Supplementary Figure 1*A*).

**Figure 1.**
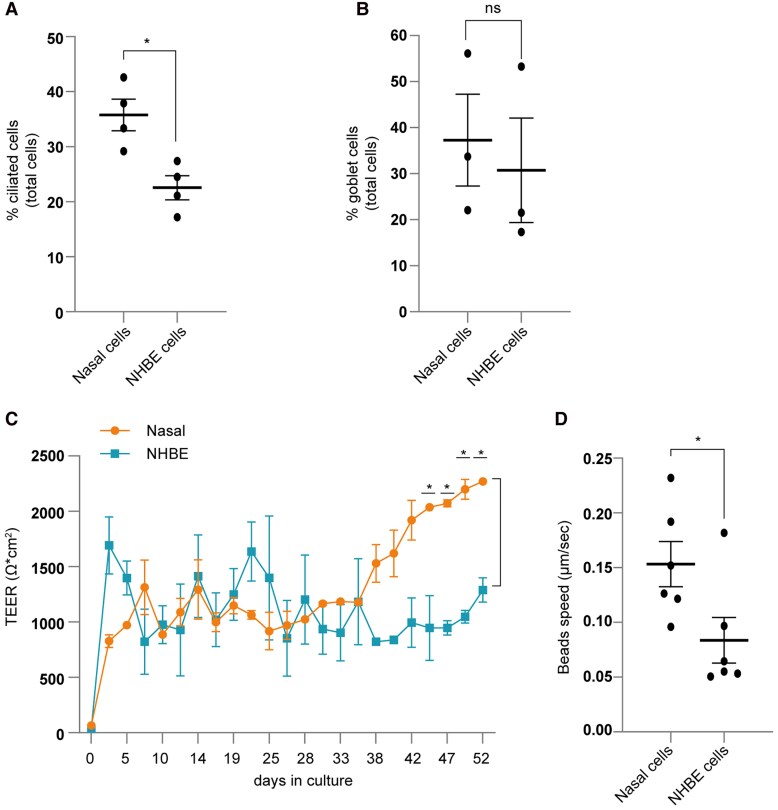
Exploring intrinsic differences between air-liquid interface (ALI)–cultured human nasal and bronchial epithelial cells. *A* and *B*, Immunofluorescence quantification of ciliated cells (acetylated tubulin) (*A*) and goblet cells (MUC5AC) (*B*). *C*, Tissue integrity was analyzed by transepithelial electrical resistance (TEER) measurements of nasal and normal human bronchial epithelial (NHBE) cells over a period of 52 days. *D*, Mucociliary clearance rates were measured by monitoring the transport of fluorescently labeled beads across the nasal and NHBE ALI cultures. Data show the mean values and error bars are the standard error of the mean. *A* and *B*, Unpaired *t*-test: *P ≤ .05 (n = 4 donors). *C*, Two-way analysis of variance with Šídák multiple comparisons test: *P ≤ .05 (n = 4 donors measured in duplicate). *D*, Unpaired *t*-test: *P ≤ .05 (n = 6 donors). ns, nonsignificant.

IF analyses revealed higher percentages of ciliated cell levels and goblet cells in nasal epithelial cells compared to NHBE cells (Figure 1*A* and 1*B*). Barrier integrity was assessed via TEER measurement [9] on Transwell inserts (Figure 1*C*). TEER remained similar in nasal and NHBE cultures for 5 weeks of differentiation, but from day 40 onward, nasal cells showed significantly higher TEER (Figure 1*C*) and demonstrated higher ciliary beat frequency than NHBE cells (Figure 1*D*). Thus, while nasal and NHBE ALI cultures shared common features, they differed in cell composition and ciliary motility.

### Comparison of Influenza A and Influenza B Virus Infection Kinetics in Nasal and Upper Bronchial Epithelial Cells

**Figure 2.**
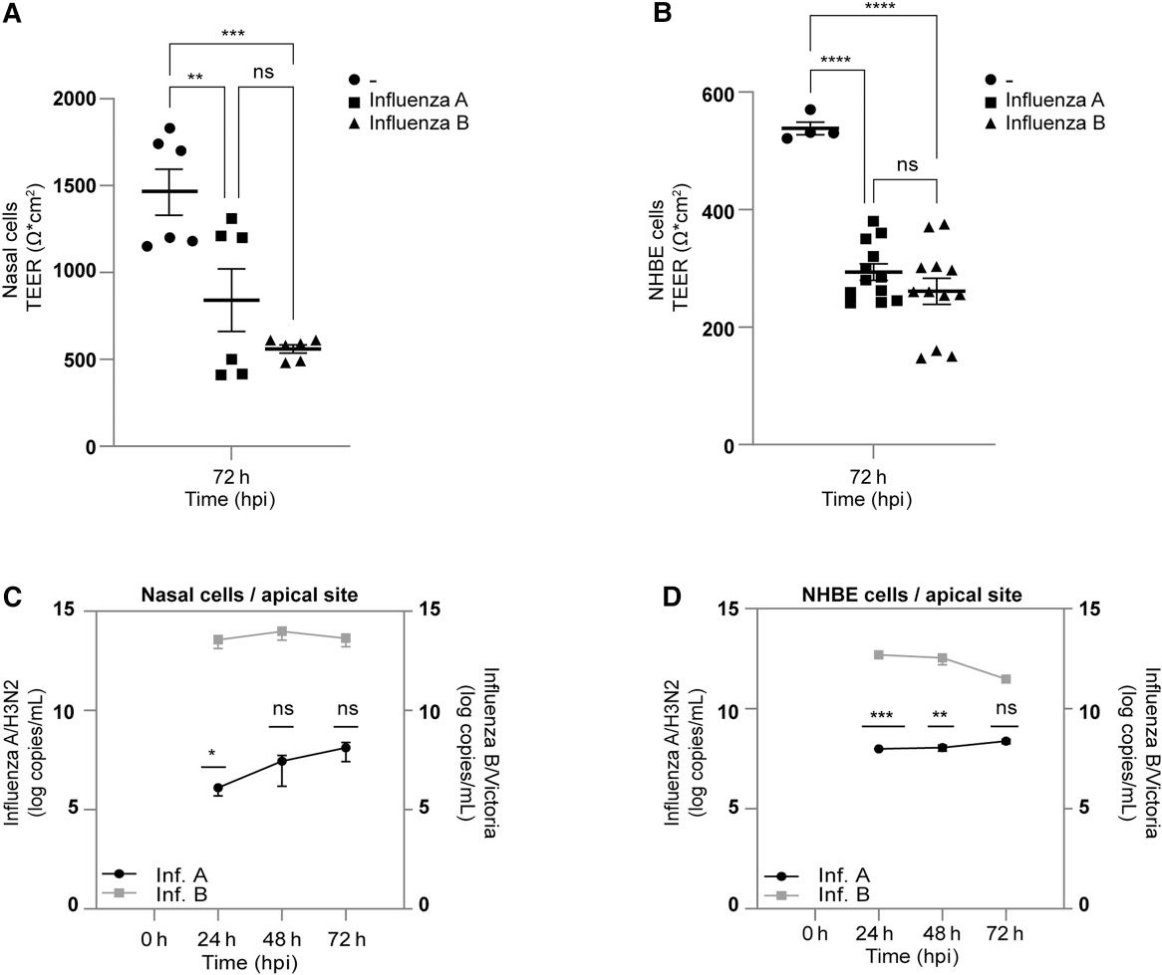
Disruption of epithelial integrity by influenza A/H3N2 and influenza B/Victoria infection of nasal and normal human bronchial epithelial (NHBE) cells. Influenza A/H3N2 and influenza B/Victoria damage the *in vitro* airway epithelium. *A* and *B*, Pseudostratified nasal (*A*) and NHBE (*B*) epithelia were infected apically by influenza A/H3N2 and influenza B/Victoria. Transepithelial electrical resistance (TEER) measurements were analyzed at 72 hours postinfection (hpi). TEER values (Ω*cm^2^) were determined for all conditions, unstimulated and infected (influenza A/H3N2 and influenza B/Victoria). *C* and *D*, Apical viral RNA (log copies/mL) from influenza A/H3N2– and influenza B/Victoria–infected epithelia was analyzed from the supernatants at 24, 48 and 72 hpi. Data show the mean values and error bars are the standard error of the mean. *A* and *B*, Ordinary one-way analysis of variance (ANOVA) with Tukey multiple comparisons test: **P ≤ .01, ***P ≤ .001, ****P < .0001 (*A*, n = 3 donors in duplicate; *B*, n = 6 donors in duplicate). *C* and *D*, Two-way ANOVA with Tukey multiple comparisons test: *P ≤ .05, **P ≤ .01, ***P ≤ .001 (n = 5 donors). ns, nonsignificant.

Next, we evaluated susceptibility of nasal and NHBE cells to influenza A/H3N2 and B/Victoria viruses by infecting them at a multiplicity of infection of 0.01 and monitoring replication kinetics. Influenza viruses were inoculated apically, and TEER was measured during the course of infection (Figure 2*A* and 2*B*). Viral load was measured every 24 hours by qPCR in apical washes (Figure 2*C* and 2*D*) and in basolateral medium (Supplementary Figure 1*D* and 1*E*). Consistent with previous reports, both viruses infected cells in nasal and bronchial ALI tissues at 24 hpi, followed by a gradual decline (Figure 2*C* and 2*D*). Viral RNA appeared in the basolateral medium at 48 hpi (Supplementary Figure 1*D* and 1*E*), indicating a barrier breach by 48 hpi and 72 hpi consistent with TEER drops (Figure 2*A* and 2*B*). Thus, both influenza A/H3N2 and B/Victoria effectively infected and replicated in nasal and NHBE cells. Additionally, we tested the neuraminidase inhibitor DANA, which exhibits inhibitory activity against various human neuraminidase enzymes (NEU1–4) involved in viral pathogenesis [26]. DANA significantly reduced viral replication, as demonstrated by lower viral RNA levels of NHBE cells when compared to untreated controls (Supplementary Figure 1*F*). This reduction was particularly pronounced in the case of influenza B/Victoria infection, which aligns with previous findings in which neuraminidase synthesizes its own inhibitors, making it highly susceptible to DANA [27]. While neuraminidase inhibitors are primarily known for blocking viral egress, recent studies suggest that neuraminidase also facilitates viral entry by modulating receptor availability and enabling viral movement along the cell surface [28, 29]. Influenza A/H3N2 and B/Victoria binding to NHBE cells was reduced in the presence of enoxaparin or DANA (Supplementary Figure 2*A* and 2*B*), indicating that neuraminidase inhibition by DANA may also impair virus–cell binding.

### Replication of Influenza Viruses Is Inhibited by Enoxaparin

**Figure 3.**
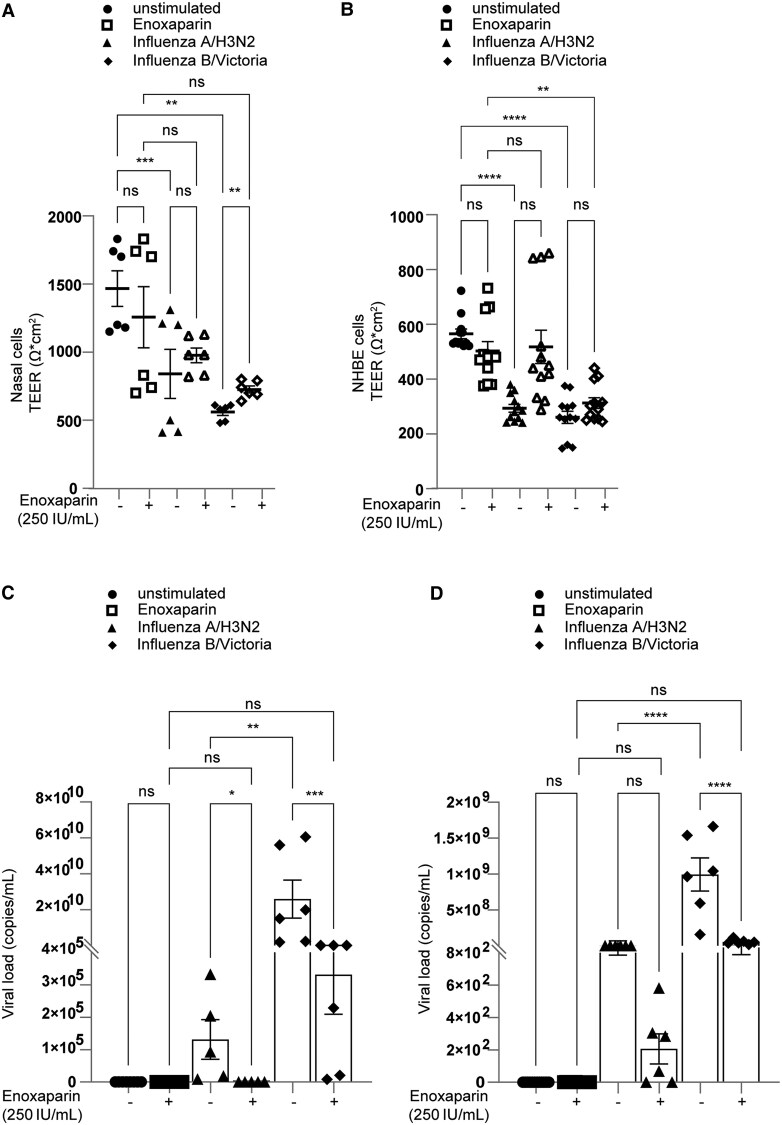
Influenza A/H3N2 and influenza B/Victoria infection of polarized nasal and normal human bronchial epithelial (NHBE) cells and tissue disruption was inhibited by the low-molecular-weight heparin, enoxaparin. Pretreatment with enoxaparin protects airway epithelia from tissue disruption and influenza infection. *A* and *B*, Polarized nasal (*A*) and NHBE (*B*) cells in the epithelial 3D model were exposed to enoxaparin (250 IU/mL) via nebulization and subsequently infected with influenza A/H3N2 and influenza B/Victoria (multiplicity of infection, 0.01). Tissue integrity was monitored by transepithelial electrical resistance (TEER; Ω*cm^2^) measurements at 72 hours postinfection using EVOM volt ohmmeter. *C* and *D*, Influenza A/H3N2 and influenza B/Victoria infections were measured by reverse-transcription quantitative polymerase chain reaction (qPCR) of viral RNA after 72 hours. To emphasize biologically meaningful differences, viral load data were normalized to the mean signal observed in the uninfected + enoxaparin condition. The low-level signals detected in uninfected samples (± enoxaparin) reflect background amplification intrinsic to the qPCR assay and are several orders of magnitude lower than the values measured in infected samples. Data show the mean values and error bars are the standard error of the mean. *B* and *C*, Repeated measures one-way analysis of variance (ANOVA) (matched donors), followed by Tukey multiple comparisons test. *B*, **P ≤ .01, ***P ≤ .001 (n = 3 donors in duplicate). *C*, *P* < .0001 (n = 6 donors in duplicate). *C* and *D*, Ordinary one-way ANOVA with Tukey multiple comparisons test. *C*, *P ≤ .05, **P ≤ .01, ***P ≤ .001 (n = 6 donors). *D*, ****P < .0001 (n = 6 donors). ns, nonsignificant.

The nasal epithelium abundantly expresses HSPGs [30] that facilitate viral attachment [14, 16]. We investigated the expression of HSPG–Syndecan 1 and 4 transcripts in the nasal and bronchial ALI tissues. Both ALI tissues expressed Syndecan 1 and 4, with NHBE cells showing higher mRNA levels of each (Supplementary Figure 2*C*). Following cellular composition characterization, ALI tissues were exposed to enoxaparin prior to infection with influenza A/H3N2 or B/Victoria, given the known role of HSPG in viral attachment [14, 16]. TEER measurements revealed that, despite enoxaparin pretreatment, barrier function declined significantly by 72 hpi compared to uninfected controls. Nevertheless, enoxaparin preserved epithelial integrity in influenza A/H3N2–infected NHBE cells up to 72 hpi (with a similar trend in nasal cells), but failed to protect integrity during influenza B/Victoria infection in either nasal or bronchial cells (Figure 3*A* and 3*B*).

Although enoxaparin preserved tissue integrity in NHBE cells following influenza A/H3N2 infection for up to 3 days, a notable decrease in viral loads for both influenza viruses was observed. This reduction, albeit to a lower level, occurred in nasal (Figure 3*C*) and NHBE (Figure 3*D*) tissues that were pretreated with enoxaparin prior to viral exposure.

IF analysis confirmed reduced fluorescence in enoxaparin-treated cells (Supplementary Figure 2*E* and 2*F*), compared to controls (Supplementary Figure 2*D*), indicating a dose-dependent inhibition of influenza replication. At 250 IU/mL, influenza NP staining was particularly diminished in influenza B/Victoria–infected cells (Supplementary Figure 2*E* and 2*F*), highlighting enoxaparin concentration-dependent antiviral effect. Furthermore, enoxaparin exhibited IC_50_ of 126.4 IU/mL for influenza A/H3N2, 1.016 IU/mL for influenza B/Victoria, and 4.191 IU/mL for SARS-CoV-2 WT, providing a quantitative comparison of its antiviral potency against these viruses (Supplementary Figure 3*A*).

### Influenza Virus Infection of Nasal and NHBE Tissues Results in Significant Cytotoxicity

**Figure 4.**
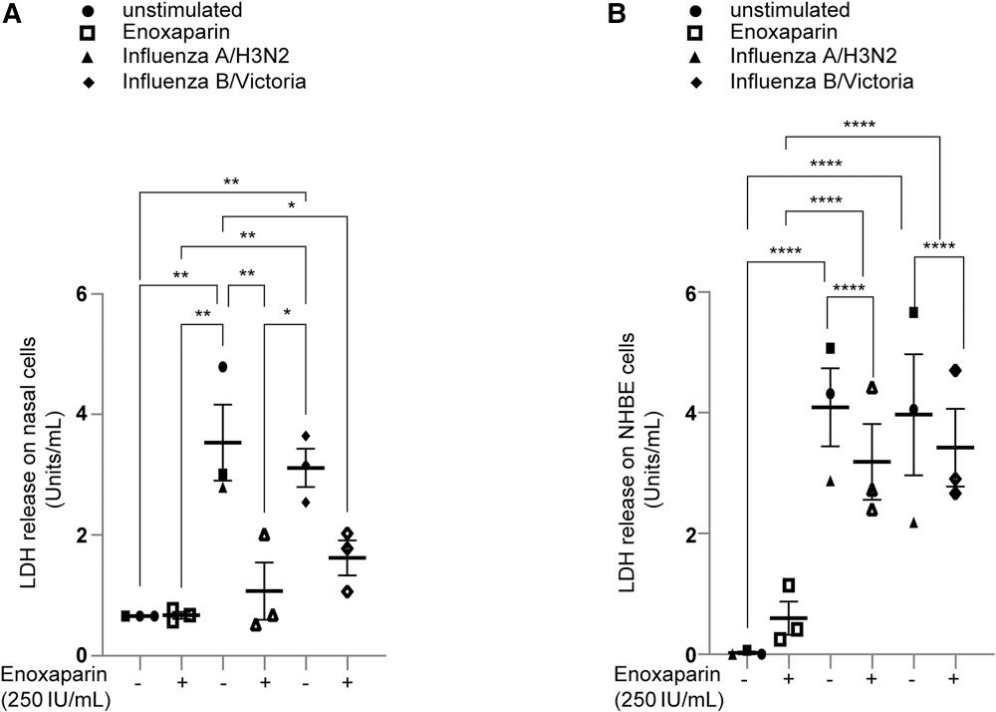
Influenza-mediated cytotoxicity was blocked in nasal and normal human bronchial epithelial (NHBE) cell cultures by enoxaparin pretreatment. Polarized nasal and NHBE cells were pretreated with enoxaparin (250 IU/mL) and subsequently infected with influenza A/H3N2 and influenza B/Victoria (multiplicity of infection, 0.01). *A* and *B*, Assessment of cell viability after 72 hours postinfection through measurement of lactate dehydrogenase (LDH) release in the supernatants of nasal (*A*) and NHBE (*B*) cells. Data show the mean values and error bars are the standard error of the mean. *A* and *B*, Two-way analysis of variance with Tukey multiple comparisons test. *A*, *P ≤ .05, **P ≤ .01 (n = 3 donors). *B*, ****P < .0001 (n = 3 donors).

Next, we investigated whether enoxaparin could rescue cells from virus-induced cell damage. At 72 hpi, apical supernatants from influenza A/H3N2 or B/Victoria-infected nasal (Figure 4*A*) and NHBE (Figure 4*B*) cultures were analyzed for LDH release as a marker of cell membrane integrity loss (ie, cytotoxicity). Both viruses significantly induced LDH release in nasal and NHBE cultures (Figure 4*A* and 4*B*, respectively), consistent with virus-induced cytopathic effects and associated cell damage. Enoxaparin pretreatment significantly reduced LDH release in nasal cultures (Figure 4*A*), and a smaller, but measurable, reduction in NHBE (Figure 4*B*, Supplementary Figure 3*B*) cultures, indicating a partial protection against virus-induced cytotoxicity. Additionally, comparison with SARS-CoV-2 infection [14, 16] confirmed a similar cytotoxic profile, which was also mitigated by enoxaparin (Supplementary Figure 3*C*), supporting its direct antiviral protection. To assess potential cytotoxicity of enoxaparin itself, LDH release was measured at 30 minutes, 4 hours, 16 hours, and 24 hours posttreatment (250IU/mL) in NHBE cells. LDH peaked at 30 minutes posttreatment, indicating a low and temporary cytotoxic effect that diminished over time (Supplementary Figure 3*D*).

### Enoxaparin Dampens Cytokine Release of Airway Epithelia Upon Influenza Infection

**Figure 5.**
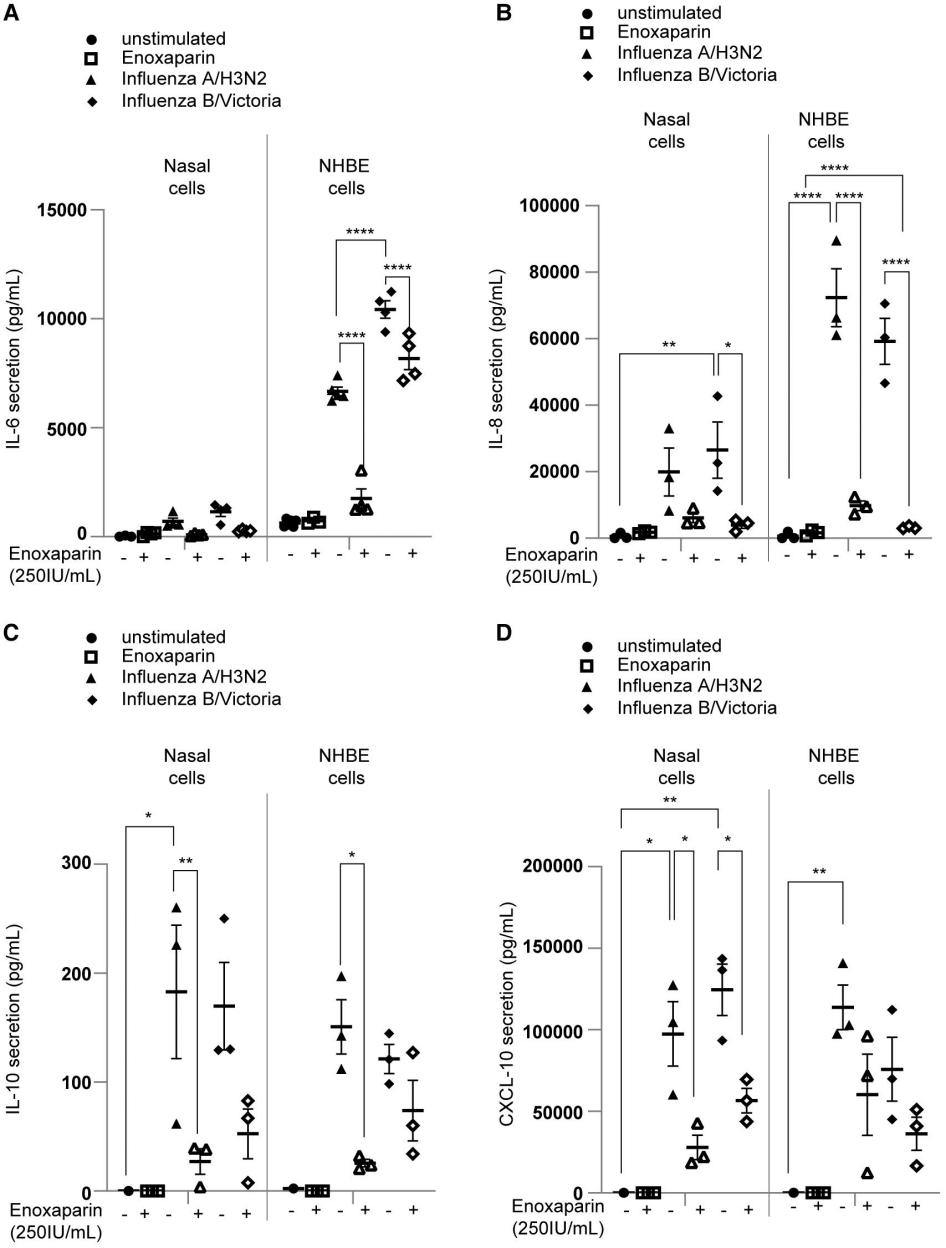
Influenza-induced inflammation was blocked by enoxaparin pretreatment in nasal and normal human bronchial epithelial (NHBE) cultures. Polarized nasal and NHBE cells were pretreated with enoxaparin (250 IU/mL) and then infected with influenza A/H3N2 or influenza B/Victoria (multiplicity of infection, 0.01). Inflammatory responses were measured by analyzing cytokine and chemokine secretion. The levels of IL-6 (*A*), IL-8 (*B*), IL-10 (*C*), and CXCL-10 (*D*) were measured by enzyme-linked immunosorbent assay in the supernatant of nasal and NHBE cells 72 hours postinfection. Data show the mean values from 3 independent experiments performed in duplicate and error bars are the standard error of the mean. *A–D*, Two-way analysis of variance with Tukey multiple comparisons test. *A*, ****P < .0001 (nasal cells, n = 3 donors; NHBE cells, n = 4 donors). *B*, *P ≤ .05, **P ≤ .01, ****P < .0001 (n = 3 donors). *C*, *P ≤ .05, **P ≤ .01 (n = 3 donors). *D*, *P ≤ .05, **P ≤ .01 (n = 3 donors).

Cytokine and chemokine secretion in influenza-infected ALI tissues was quantified by ELISA. Both influenza A/H3N2 and B/Victoria significantly increased IL-6, IL-8, IL-10, and CXCL-10 secretion compared to uninfected/untreated and enoxaparin-treated controls (Figure 5*A*–*D*). NHBE cells released more IL-6 and IL-8 than nasal cells (Figure 5*A* and 5*B*), indicating distinct immune profiles between upper and lower airways. These differences suggest intrinsic disparities in early responses to influenza viruses between the upper and lower respiratory tracts.

To assess therapeutic potential, enoxaparin was applied 24 hpi. Unlike enoxaparin pretreatment, delayed administration did not reduce IL-10, IL-8, or IL-6 (Supplementary Figure 4*A–C*), underscoring the importance of treatment timing for its protective and anti-inflammatory effects.

### Enoxaparin Protects Human Airway Epithelial Cells From Intracellular C3 Mobilization

**Figure 6.**
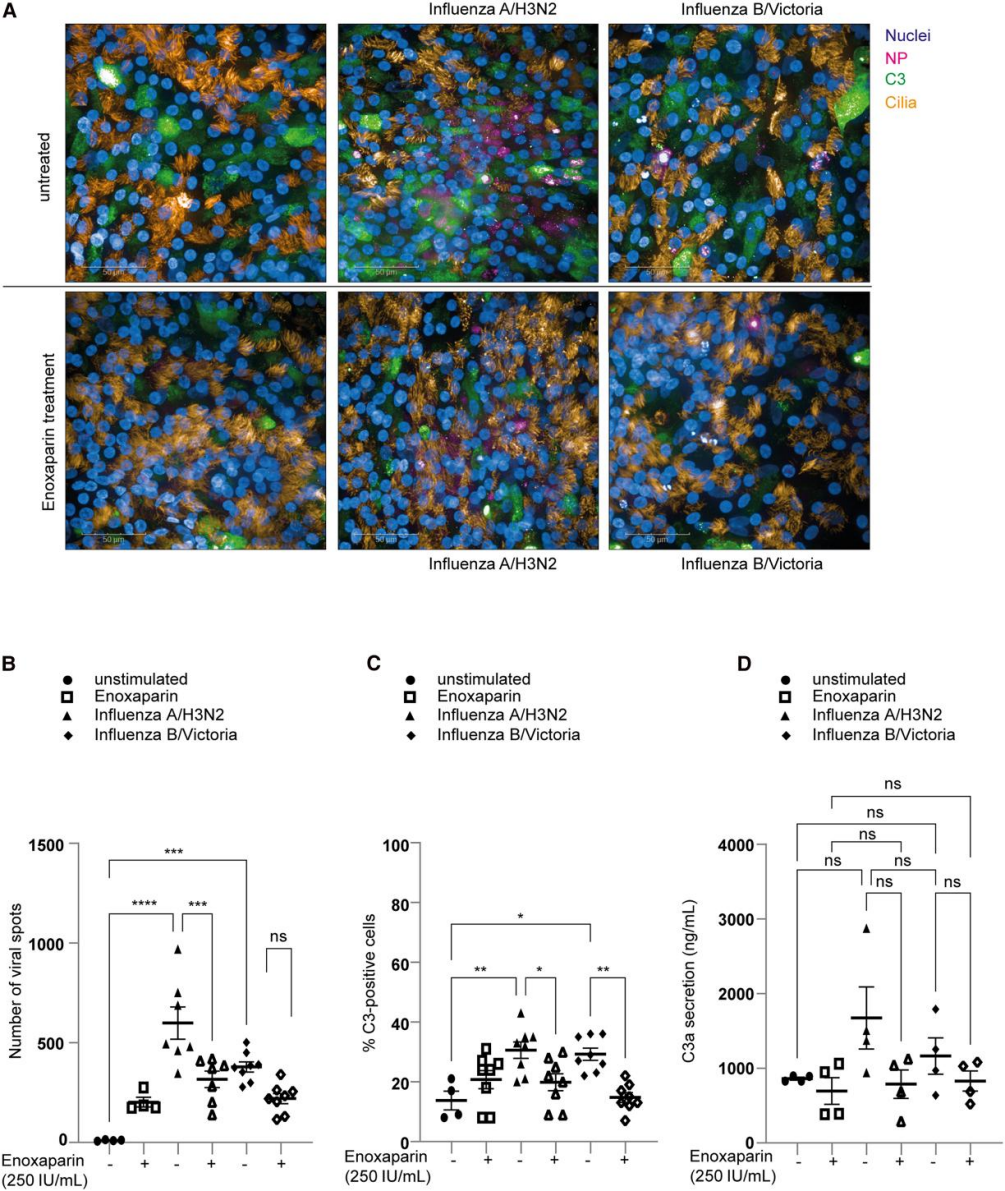
The low-molecular-weight heparin enoxaparin prevents polarized nasal cells from influenza A/H3N2 and influenza B/Victoria infection and immune activation. Pseudostratified nasal epithelia were grown on Transwells for at least 60 days and treated apically with enoxaparin prior exposure to influenza A/H3N2 and influenza B/Victoria. At 48 hours postinfection, filters were fixed and stained for relevant markers. Nuclei (Höchst, blue), influenza nucleoprotein (nucleoprotein [NP], pink), complement C3 (green), and acetylated tubulin (cilia, orange) are illustrated. *A*, Representative immunofluorescence images (63× water magnification) of apical junction complex proteins of epithelial cells. Visualization of nasal epithelia together with virus binding (influenza NP, Alexa 594), C3-FITC as indicator of immune activation, and acetylated tubulin for staining cilia. Scale bars represent 50 µm. *B* and *C*, Number of viral spots (*B*) and % C3-positive cells (*C*) were absolutely quantified from at least 3 different areas using Harmony 4.9 software. Notably, the weak signal observed in the uninfected, enoxaparin-treated condition (*B*, left lane) likely reflects autofluorescence or nonspecific background staining rather than actual viral presence, a phenomenon occasionally seen in primary nasal epithelial cells. *D*, C3a production levels were determined by enzyme-linked immunosorbent assay. Data show the mean values and error bars are the standard error of the mean. *B–D*, Ordinary one-way analysis of variance with Tukey multiple comparisons test. *B*, ***P ≤ .001, ****, *P* < .0001 (n = 4 donors in duplicate). *C*, *P ≤ .05, **P ≤ .01 (n = 4 donors in duplicate). *D*, n = 4 donors. ns, nonsignificant.

**Figure 7.**
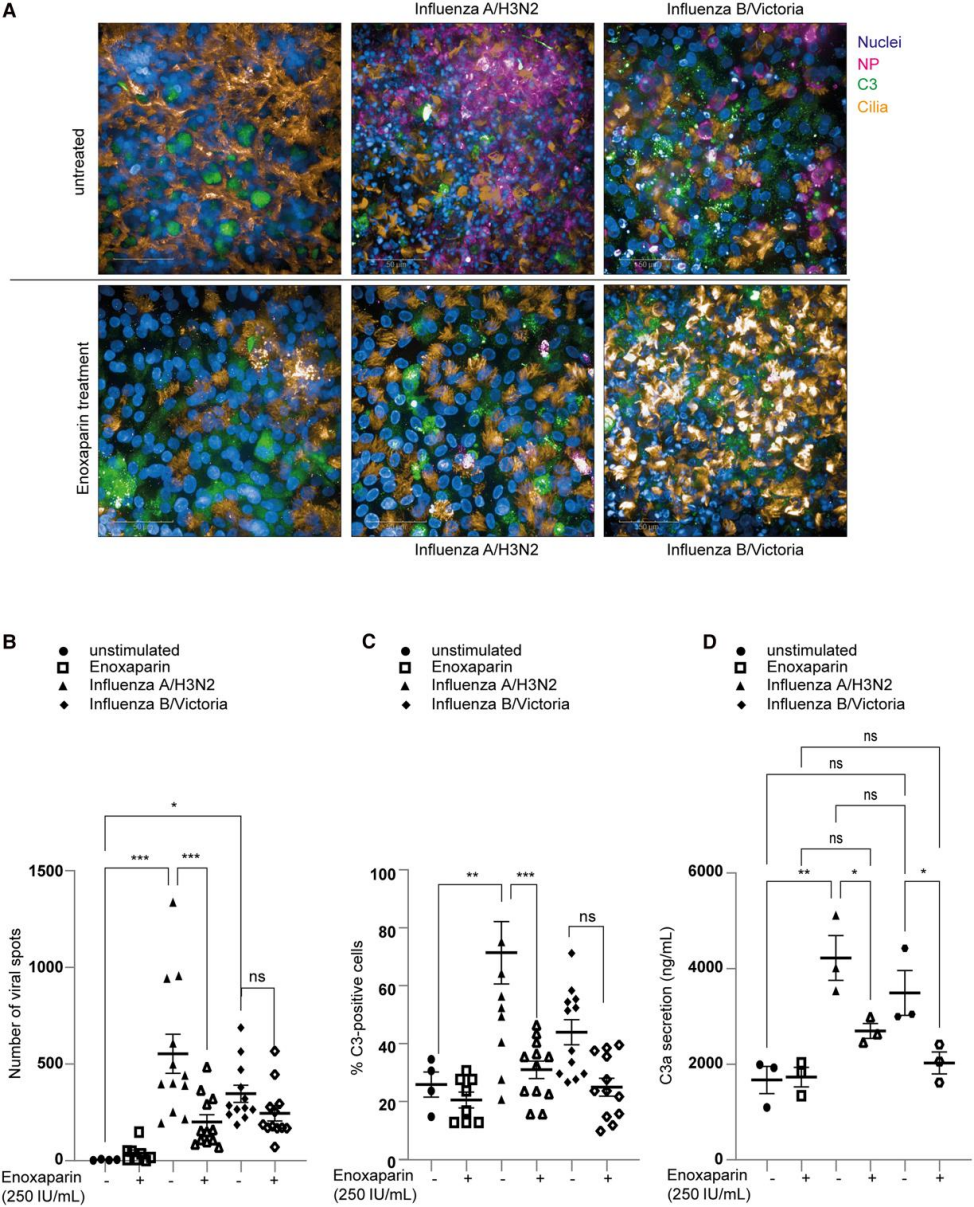
The low-molecular-weight heparin enoxaparin prevents polarized normal human bronchial epithelial (NHBE) cells from influenza A/H3N2 and influenza B/Victoria infection and immune activation. Pseudostratified bronchial epithelia were grown on Transwells for at least 60 days and treated apically with enoxaparin prior exposure to influenza A/H3N2 and influenza B/Victoria. At 48 hours postinfection, filters were fixed and stained for relevant markers. Nuclei (Höchst, blue), influenza nucleoprotein (NP, pink), complement C3 (green), and acetylated tubulin (cilia, orange) are depicted. *A*, Representative immunofluorescence images (63× water magnification) of apical junction complex proteins of epithelial cells. Visualization of bronchial epithelia together with virus binding (influenza NP, Alexa 594), C3-FITC as indicator of immune activation, and acetylated tubulin for staining cilia. Scale bars represent 50 µm. *B* and *C*, Number of viral spots (*B*) and % C3-positive cells (*C*) were absolutely quantified from at least 3 different areas using Harmony 4.9 software. *D*, C3a production levels were determined by enzyme-linked immunosorbent assay. Data show the mean values and error bars are the standard error of the mean. *B–D*, Ordinary one-way analysis of variance with Tukey multiple comparisons test. *B*, *P ≤ .05, ***P ≤ .001 (n = 6 donors in duplicate). *C*, **P ≤ .01, ***P ≤ .001 (n = 4 donors in duplicate). *D*, *P ≤ .05 (n = 3 donors). ns, nonsignificant.

Given our recent data on local complement induction upon influenza A/H3N2 and influenza B/Victoria challenge in NHBE cultures [22], we analyzed the impact of enoxaparin on complement activation in nasal and NHBE cells upon both influenza viruses (Figures 6 and 7). Intracellular C3 served as an innate immune activation marker during viral infection (Figures 6 and 7) [22]. Strikingly, both nasal and NHBE cells illustrated strong influenza A/H3N2 uptake (Figures 6*B* and 7*B*), whereas influenza B/Victoria was moderately taken up by NHBE compared to nasal cells. Consistent with previous findings, IF revealed that enoxaparin reduced viral presence in both ALI tissues (fewer virus spots) and prevented the increase of intracellular C3 signal (Figures 6*B*  6*C*, 7*B*, and 7*C*). Weak background staining in uninfected, enoxaparin-treated nasal cells (Figure 6*B*) reflected autofluorescence occasionally seen in primary epithelial cultures. Together, these findings suggested that enoxaparin attenuates viral replication and dampens host inflammatory response to influenza infection. Nevertheless, our data showed that influenza A/H3N2 caused stronger effects than influenza B/Victoria on nasal cells (Figure 6*A*–*D*) and NHBE cells (Figure 7*A*–*D*), evidenced by higher viral loads and increased LDH and cytokine/chemokine production. Strikingly, the most significant reductions in viral load and C3 mobilization occurred in influenza A/H3N2–infected cells. Given that influenza infection triggered C3 mobilization, also recently described for SARS-CoV-2 [31] and influenza [22], we measured C3a and C5a levels on the basolateral subnatants. Consistent with IF results, C3a levels were significantly higher in influenza-infected cultures than in uninfected, uninfected + enoxaparin, and enoxaparin/virus cultures (Figures 6*D* and 7*D*). These results support enoxaparin’s protective role in preserving epithelial integrity, reducing viral replication, and preventing excessive complement-driven inflammation, consistent with previous findings in SARS-CoV-2 [31] and influenza [22] as well as with our data observed in SARS-CoV-2–infected NHBE cultures (Supplementary Figure 4*D* and 4*E*).

## DISCUSSION

Viral respiratory infections are a major global health burden, causing lung damage, secondary infections, and subsequent chronic respiratory diseases. Understanding these infectious pathways is crucial for developing effective antiviral strategies. Our research demonstrated that primary human nasal and bronchial ALI tissues provide reliable 3D in vitro tissue models for studying infection dynamics and their impacts on the respiratory tract, specifically by simulating immune responses.

Airborne pathogens like influenza and SARS-CoV-2 enter through the respiratory tract, making them a key target for early intervention. While vaccination remains essential and effective, optimal protection requires immune responses at mucosal surfaces. Research on nasal vaccines has suggested that they may not generate an equally robust immune defense (lower hemagglutination inhibition geometric mean titers) compared to conventional vaccines [32, 33]. This underscores the need for alternative products to enhance prevention against respiratory pathogens.

Recent studies showed that LMWHs possess anti-inflammatory effects [34–36] and reduce viral attachment by competing with heparan sulfate [13, 14, 37–39]. Enoxaparin has demonstrated promising protective effects against pathogenic viruses [14, 16, 20, 40], without compromising its safety profile in anticoagulation [16 , 18]. We previously demonstrated its safety as a preventive therapeutic [16]. Notably, HSPG expression may vary with age, comorbidities (eg, diabetes, chronic obstructive pulmonary disease), and airway inflammation, potentially modulating the antiviral activity of enoxaparin. Thus, while our findings in healthy epithelial cells are promising, outcomes may differ in patient populations with impaired epithelial integrity, chronic inflammation, or compromised mucosal barriers. For example, elderly individuals or patients with chronic lung diseases may exhibit reduced HSPG availability, impacting drug–target interactions. Future studies in these populations are required to better define the therapeutic potential and limitations of enoxaparin.

We investigated the antiviral properties of enoxaparin against influenza using 2 highly differentiated respiratory 3D models. Nasal epithelial cells exhibit higher MUC5AC expression, greater abundance of ciliated and goblet cells, and increased TEER and cilia beat frequency compared to NHBE cells, confirming distinct cell composition and motility. We further examined the infection kinetics of influenza A/H3N2 and B/Victoria, finding that both viruses rapidly infected both cell types, declining thereafter. Interestingly, influenza B/Victoria showed a higher infection rate compared to influenza A/H3N2 in both nasal and bronchial epithelial cells, potentially explaining their different incubation periods. The neuraminidase inhibitor DANA significantly reduced viral replication in bronchial cells, confirming previous observations [41].

Enoxaparin markedly reduced viral loads of both influenza A/H3N2 and B/Victoria in nasal and bronchial tissues, indicating decreased transmission potential. However, influenza B/Victoria was comparatively less sensitive to enoxaparin, possibly reflecting distinct receptor interactions (eg, sialic acid), or lower-reliance HSPG-mediated entry [42]. A single enoxaparin administration only partially preserved tissue integrity by day 3 postinfection, but it rescued cells from virus-induced cytotoxicity at earlier time points, highlighting its potential as an early intervention. Repeated dosing may enhance tissue protection and warrants further investigation.

Influenza infection increased IL-6, IL-8, IL-10, and CXCL-10 secretion, consistent with previous reports [43]. Bronchial epithelial cells produced higher cytokine levels than nasal cells, indicating a stronger inflammatory response. Notably, enoxaparin significantly reduced chemokine and cytokine levels, suggesting protection against excessive immune response.

Previous studies reported that lung epithelial cells can locally activate complement (C3) and produce anaphylatoxins, with C3 secretion apically polarized to support host defense at the ALI tissue [22, 44]. In our 3D nasal and bronchial models, influenza infection induced intracellular C3 production and C3a release. Enoxaparin treatment preserved tissue integrity, reduced viral replication, and decreased C3 mobilization together with C3a release. These findings suggest that enoxaparin limits viral entry, thereby reducing complement activation and inflammation. While enoxaparin has been reported to possess intrinsic anti-inflammatory properties [45, 46], our data primarily indicate an indirect antiviral action that diminishes the need for extensive immune activation. This aligns with reports from the coronavirus disease 2019 pandemic, where complement activation worsened tissue injury [31, 47, 48]. Elevated C3a and C5a are also implicated in chronic lung conditions like cystic fibrosis and idiopathic pulmonary fibrosis [49, 50], as well as acute inflammation leading to tissue damage.

Our study highlights enoxaparin as a promising prophylactic for respiratory viral infection (influenza and SARS-CoV-2). Its protective effect arises from blocking infection and limiting complement activation. Findings from our 3D models support its potential *in vivo*. Further investigation is required to better understand the differences between influenza types. We hypothesize that enoxaparin counteracts the prothrombotic environment induced in infected epithelial and endothelial cells, suggesting that anticoagulants may effectively inhibit pathogenic influenza A/H3N2 infection. Given the ongoing threat of emerging respiratory viruses, these insights advance the development of broad-spectrum prophylactic strategies.

## Supplementary Material

jiaf470_Supplementary_Data

## Supplementary Data

Supplementary materials are available at *The Journal of Infectious Diseases* online (http://jid.oxfordjournals.org/). Supplementary materials consist of data provided by the author that are published to benefit the reader. The posted materials are not copyedited. The contents of all supplementary data are the sole responsibility of the authors. Questions or messages regarding errors should be addressed to the author.
