# Supplementary material for: The Role of Enoxaparin in Influenza Virus Infections and its Therapeutic Implications
Source: J Infect Dis. 2025 Sep 10;233(1):e118–31. doi: 10.1093/infdis/jiaf470 (PMC12811870; doi:10.1093/infdis/jiaf470)
Supplement: jiaf470_Supplementary_Data [file jiaf470_supplementary_data.pdf]

Suppl. Table 1:

Primers for Influenza Virus		
Influenza A (H3N2)	Forward	CCM AGG TCG AAA CGT AYG TTC TCT CTATC
	Reverse	TGA CAG RAT YGG TCT TGT CTT TAG CCAYTC CA
	Probe	Fam-ATYTCG GCT TTG AGG GGG CCT BHQ
Influenza B (Victoria)	Forward	CCT GTT ACA TCT GGG TGC TTT CCT ATA ATG
	Reverse	GTT GAT ARC CTG ATA TGT TCG TAT CCT CKG
	Probe	Fam-TTA GAC AGC TGC CTA ACC BHQ
Primers for respiratory tract markers		
GADPH	Forward	CCATGTTCGTCATGGGTGTG
	Reverse	GGTGCTAA GCAGTTGGTGGTG
Muc5AC	Forward	GGAACTGTGGGGACAGCTCTT
	Reverse	GTCACATTCCTCAGCGAGGTC
FoxJ1	Forward	GAGACAGGTTGTGGCGGATTGA
	Reverse	ACTCGTATGCCACGCTCATCTG
Krt5	Forward	GAGGAATGCAGACTCAGTGGA
	Reverse	TAGCTTCCACTGCTACCTCCG
Syndecan 1	Forward	ATCACCTTGTCACAGCAGACCC
	Reverse	CTCCACTTCTGGCAGGACTACA
Syndecan 4	Forward	AGGTGTCAATGTCCAGCACTGTG
	Reverse	AGCAGTAGGATCAGGAAGACGGC

Suppl. Table 2:

Category	Item	Source/Vendor	Catalog number	Additional Notes / Dilution /Fluorophore
Cells	HNECs (Human Nasal Epithelial cells)	Lonza		Cultured in ALI-model
	NHBEs (Normal Human Bronchial Epithelial cells	Lonza	CC-2540 S	Cultured in ALI-model
	MDCK	ATCC	CCL-34	Influenza propagation.
	Vero E6/TMPRSS2/ACE2	CFAR/NIBSC	101003	SARS-CoV-2 propagation
Viruses	SARS-CoV-2 WT	BEI Resources	NR-52281	MOI 0.01
	SARS-CoV-2 Delta (B.1.617.2)	Isolated from patient swabs		MOI 0.01
	SARS-CoV-2 Omicron BA.5	Isolated from patient swabs		MOI 0.01
	Influenza A/H3N2	NIBSC	19/292	MOI 0.01
	Influenza B/Victoria	NIBSC	20/242	MOI 0.01
Compounds	Enoxaparin	Sanofi	Clexane Forte 6000 IE	50uL =23.4IUmL-3000IU/mL
	N-acetyl-2,3-dehydro-2-deoxyneuraminic acid (DANA)	Sigma-Aldrich		10mM
Antibodies	Hoechst 33342	Cell Signaling	#4082	UV 1/5000
	C3c	Agilent Dako	#F0201	FITC 1/50
	Influenza A Nucleoprotein	Sino Biological	#40205-R063	A594 1/100
	Influenza A Nucleoprotein	Sino Biological	#40207-T62	1/1000
	Influenza B Nucleoprotein	Sino Biological	#40438-R004	A594 1/100
	Influenza B Nucleoprotein	Sino Biological	#40438-T30	1/1000
	Acetylated-tubulin	Cell Signaling	#35652	A488 1/50
	MUC5AC	Cell Signaling	#61193	A647 1/50
	Goat anti-Rabbit IgG	Thermofisher Scientific	#A-11008	A488 1/100
	Goat anti-Rabbit IgG	Thermofisher Scientific	#A-21245	A647 1/100
	Alexa Fluor® 488 Conjugation Kit (Fast) - Lightning-Link	Abcam	#ab236553	A488
	Alexa Fluor® 647 Conjugation Kit (Fast) - Lightning-Link	Abcam	#ab269823	
	Fluorescent Beads (Cell Sorting Set up Beads)	Invitrogen	#C16508	UV

Figure Legends

Supplementary Figure 1.

Nasal and bronchial (NHBE) epithelial cell cultures from healthy donors exhibited unique mucociliary characteristics. Cultures were differentiated for 35 days. (A) mRNA expression of the cell-type specific transcriptional factors FOXJ1 (ciliated cells), KRT5 (basal cells) and MUC5AC (goblet cells) in paired ALI-differentiated nasal and bronchial epithelial cells. (B-C) Representative immunofluorescence images (63x magnification) of ciliated and mucus producing cells of the nasal epithelium (B) and bronchial epithelium (C). Scale bars represent 50 µm (XY). Epithelial cells were fixed and stained for relevant markers, nuclei (Höchst), mucus/MUC5A (green) and acetylated tubulin for the cilia (red). (D-E) Viral RNA (log copies/mL) from influenza_A/H3N2 and influenza_B/Victoria infection was analyzed from subnatants from nasal and NHBE cultures at 24h, 48h and 72h post-infection. (F) Viral RNA (log copies/mL) from influenza_A/H3N2 and influenza_B/Victoria infection was analyzed from NHBE cultures at 48h post-infection in presence or absence of sialidase inhibitor. Data show mean values and error bars are the SEM. Statistical analysis were performed using (A) 2-way ANOVA with Šídák's multiple comparisons test. $^{**}p \leq 0.01$ (n=3 donors). (D-E) 2-way ANOVA with Tukey´s multiple comparison test. $^{**}p \leq 0.01$ (n=6 donors). (F) ordinary one-way ANOVA with Tukey´s multiple comparisons test. $^{**}p \leq 0.01$ (n=3 donors). ns = non-significant.

Supplementary Figure 2.

(A-B) NHBE cells were exposed to influenza_A/H3N2 (A) and influenza_B/Victoria (B) the presence or absence of enoxaparin and a sialidase inhibitor. (C) Syndecan 1 (SYND1) and Syndecan 4 (SYND4) expression by nasal and NHBE cells was detected by quantitative real-time PCR.(D-F) Fluorospot analysis of MDCK cells infected with influenza_A/H3N2 and influenza_B/Victoria (D) and in presence (F) or absence (E) of enoxaparin treatment (0,5, 1, 5, 50, 100, 250 IU/ml). Data show mean values and error bars are the SEM. Statistical analysis were performed using (A-B) 2-way ANOVA with

Dunnett’s multiple comparisons test. *$p \leq 0.05$, **$p \leq 0.01$ (A, n=3 donors;, n=4 donors). (C) 2-way ANOVA with Šídák's multiple comparisons test. *$p \leq 0.05$ (n=3 donors). (D) 2-way ANOVA with Šídák's multiple comparisons test. **$p \leq 0.01$, ****, $p < 0.0001$ (n=3 donors). ns = non-significant.

Supplementary Figure 3.

(A) Viral-dependent inhibition of influenza_A/H3N2, influenza_B/Victoria and SARS-CoV-2 WT by enoxaparin. Dose-response inhibition curves illustrating the effect of enoxaparin on influenza_A/H3N2, influenza_B/Victoria, and SARS-CoV-2 WT, with viral inhibition measured as a function of enoxaparin concentration. IC_{50} values (half-maximal inhibitory concentration) indicate the potency of enoxaparin against each virus. (B) Lactate concentrations were measured in SNs of NHBE epithelial cultures following infection with influenza_A/H3N2 and influenza_B/Victoria plus/minus enoxaparin treatment to correlate it to the LDH release (C) Cell viability was assessed after 72 hpI by analyzing LDH release in the supernatants of NHBE (C) cells infected with SARS-CoV-2 WT, Omicron (BA.5) and Delta variants. (B.1.617.2) in presence or absence of enoxaparin. (D) NHBE cultures were treated with enoxaparin (nebulization) at different time points (0.5 h, 4 h, 16 h, and 24 h) and the cytotoxicity mediated by the compound was measured by LDH release. Data show mean values and error bars are the SEM. Statistical analysis were performed using (B-C) 2-way ANOVA with Tukey´s multiple comparisons test. *$p \leq 0.05$, **$p \leq 0.01$, ****$p < 0.0001$ (n=3 donors). (D) 2-way ANOVA with Tukey´s multiple comparisons test. (n=3 donors). ns = non-significant.

Supplementary Figure 4.

(A-C) Post-infection enoxaparin treatment assay assessing cytokine responses in nasal and NHBE cells. Cytokine levels (IL-10, IL-8, and IL-6) were measured following enoxaparin administration at 24hpI. Cytokine concentrations are plotted on a log10 scale.

(D-E) C3a (D) and C5a (E) secretion were determined for both, UI and SARS-CoV-2 (WT and VoCs) infected NHBE epithelia in presence or absence of enoxaparin treatment. Data show mean values and error bars are the SEM. Statistical analysis were performed using (A-C) 2-way ANOVA ****$p < 0.0001$ ($n=3$ donors) (D) ordinary one-way ANOVA with Tukey´s multiple comparisons test. *$p \leq 0.05$ ($n=3$ donors in triplicates). (E) ordinary one-way ANOVA with Tukey´s multiple comparisons test. *$p \leq 0.05$, ****$p < 0.0001$ ($n=3$ donors). ns = non-significant.

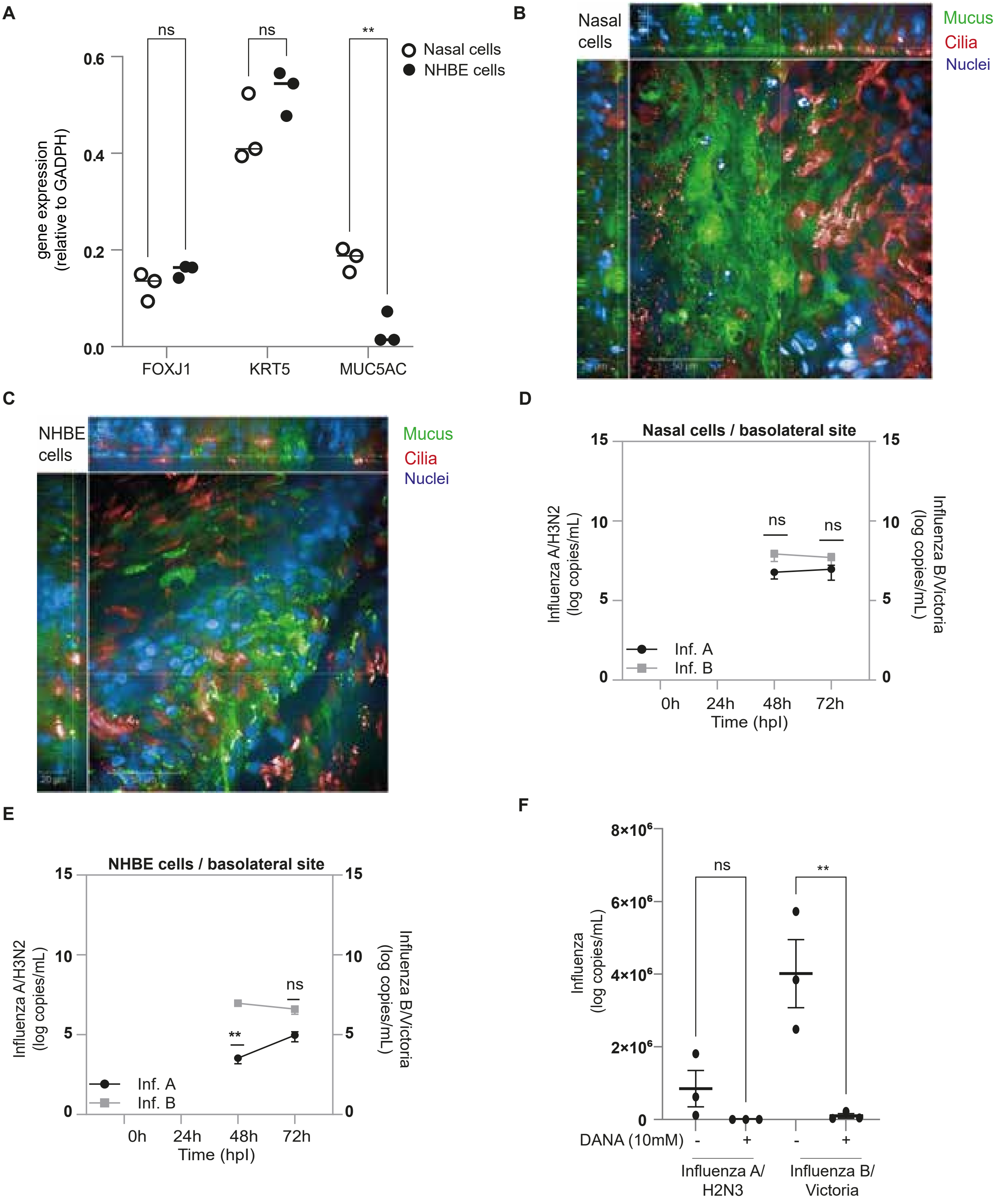

Suppl. Figure 1.

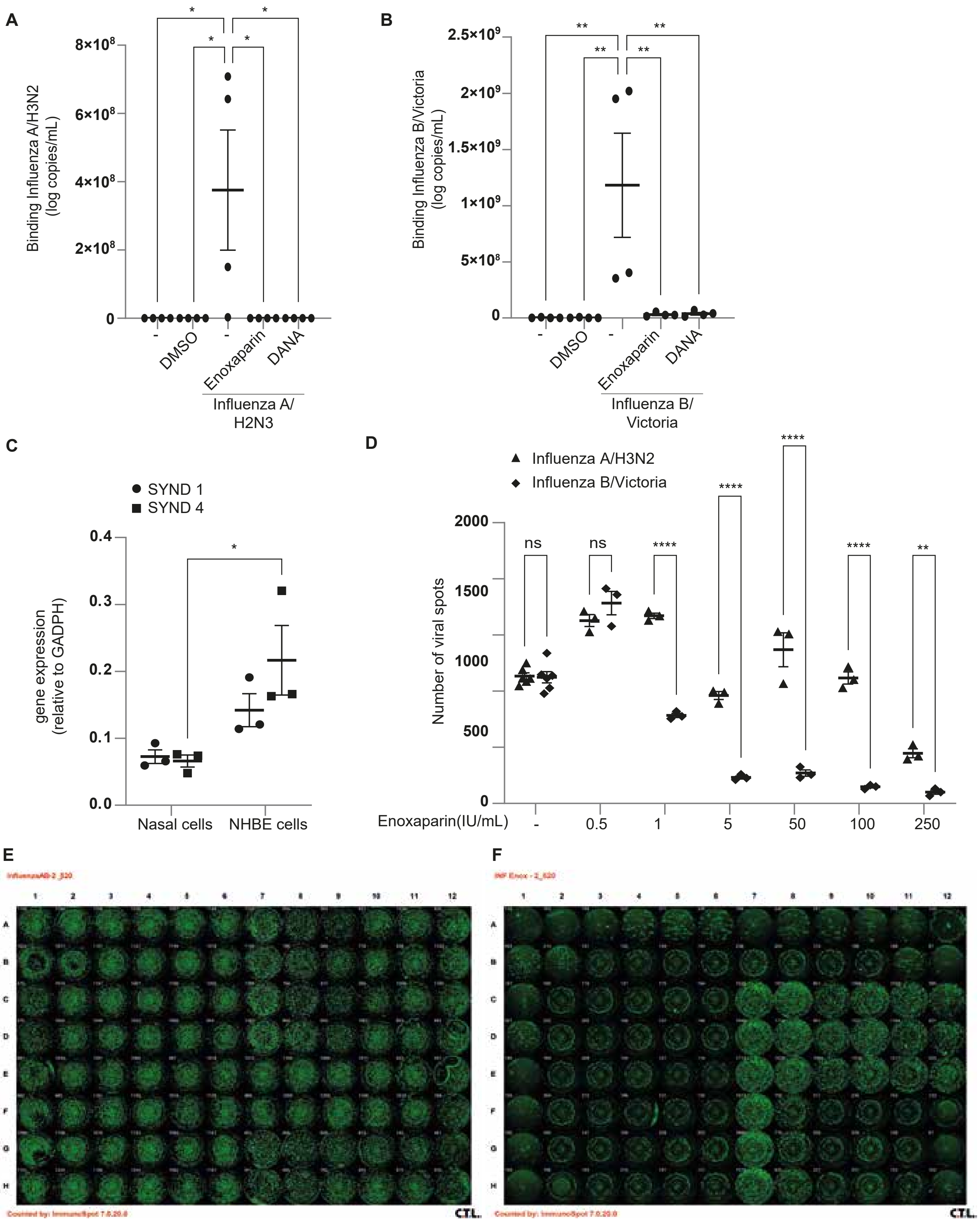

Suppl. Figure 2.

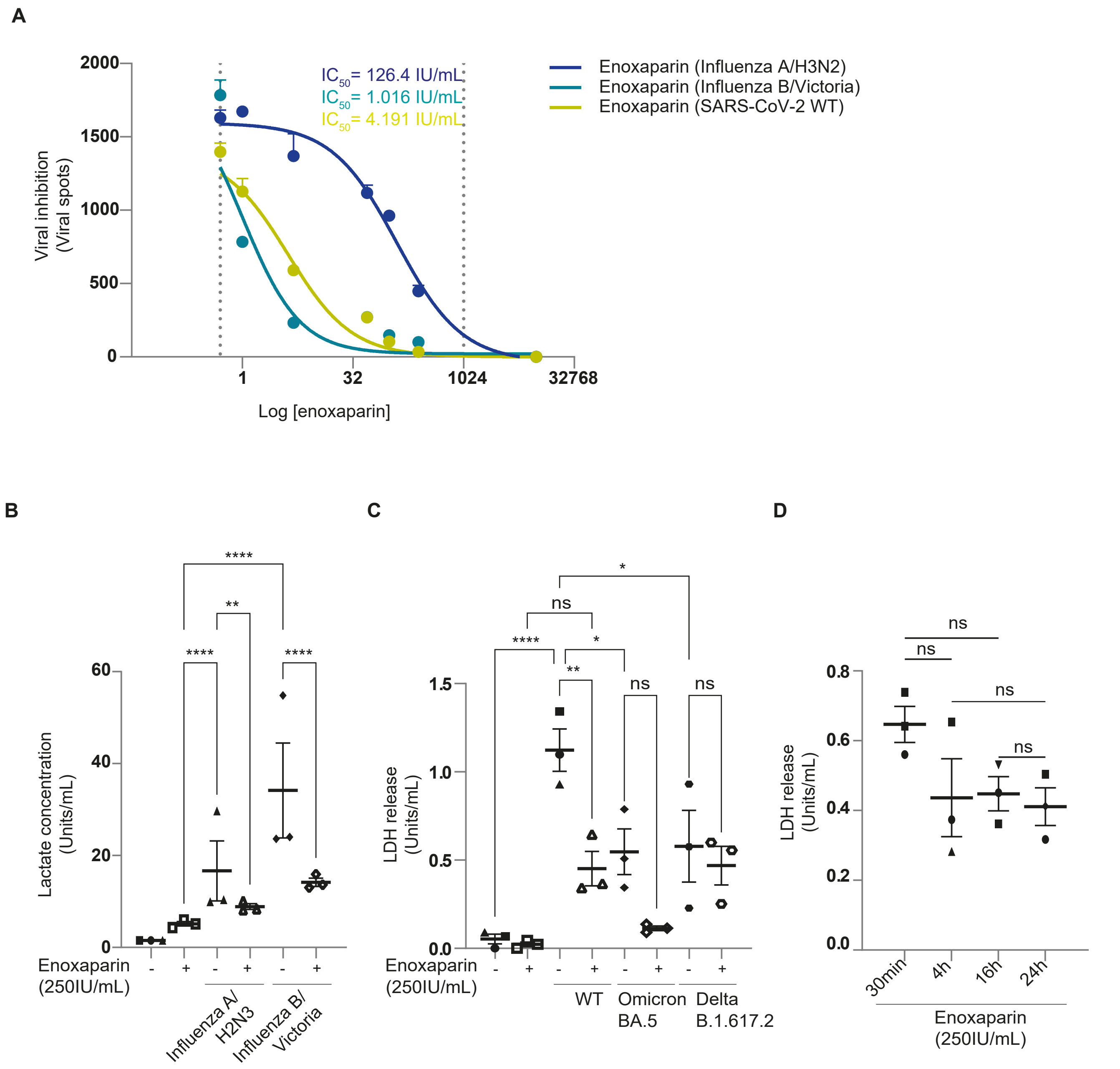

Suppl. Figure 3.

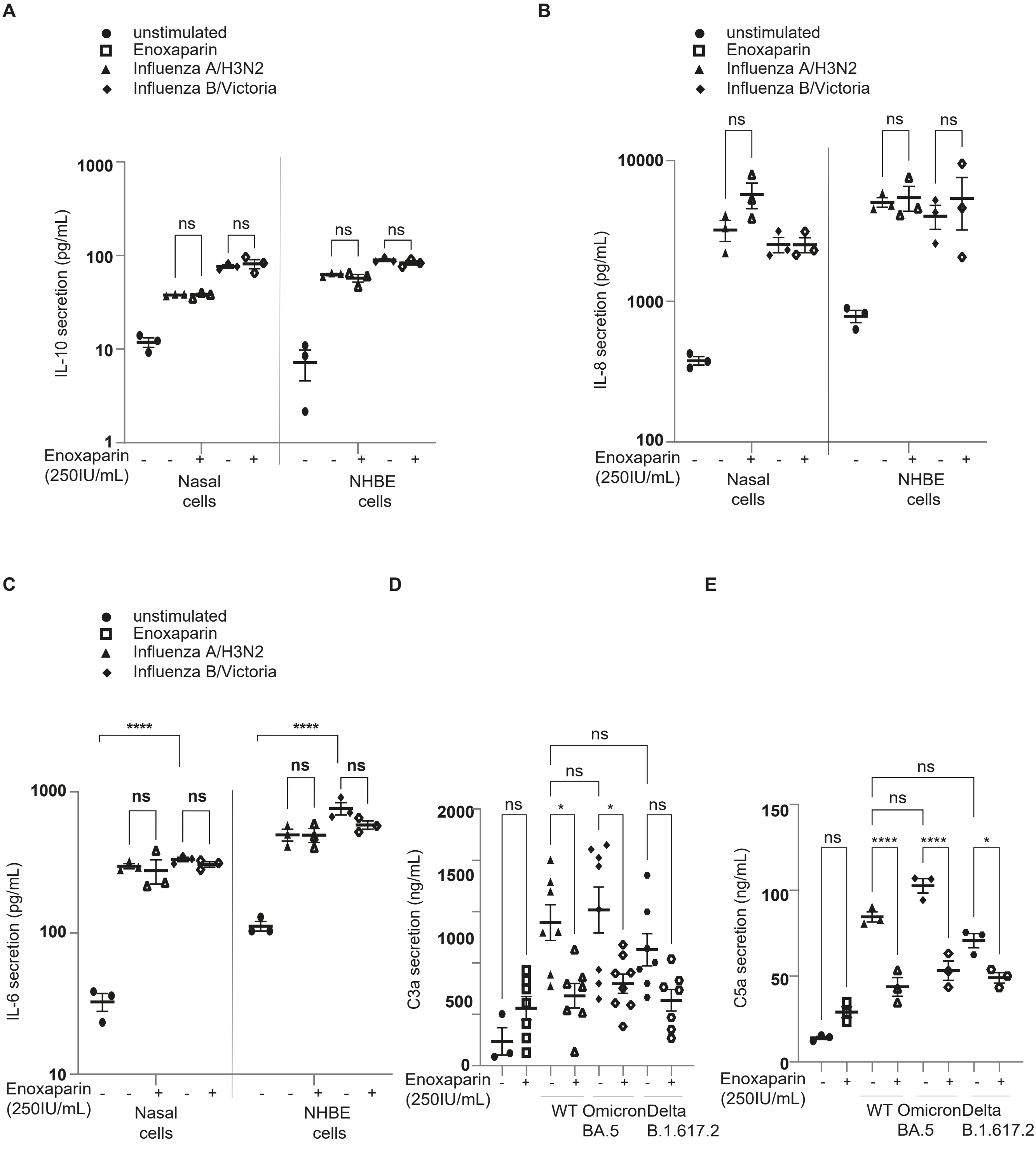

Suppl. Figure 4.
